# Data-based dynamic compartment model: Modeling of *E. coli* fed-batch fermentation in a 600 m^3^ bubble column

**DOI:** 10.1093/jimb/kuac021

**Published:** 2022-09-30

**Authors:** Jonas Bisgaard, James A Zahn, Tannaz Tajsoleiman, Tue Rasmussen, Jakob K Huusom, Krist V Gernaey

**Affiliations:** Freesense ApS, 2100 Copenhagen, Denmark; Dupont Tate & Lyle Bio Products Company, Loudon, TN 37774, USA; Freesense ApS, 2100 Copenhagen, Denmark; Freesense ApS, 2100 Copenhagen, Denmark; Process and Systems Engineering Center (PROSYS), Department of Chemical and Biochemical Engineering, Technical University of Denmark, Building 228A, 2800 Kgs. Lyngby, Denmark; Process and Systems Engineering Center (PROSYS), Department of Chemical and Biochemical Engineering, Technical University of Denmark, Building 228A, 2800 Kgs. Lyngby, Denmark

**Keywords:** Flow-following sensor devices, Compartment model, Gradients, Bubble column bioreactor, Large-scale, Mixing, Fermentation process

## Abstract

Mathematical modeling is a powerful and inexpensive approach to provide a quantitative basis for improvements that minimize the negative effects of bioreactor heterogeneity. For a model to accurately represent a heterogeneous system, a flow model that describes how mass is channeled between different zones of the bioreactor volume is necessary. In this study, a previously developed compartment model approach based on data from flow-following sensor devices was further developed to account for dynamic changes in volume and flow rates and thus enabling simulation of the widely used fed-batch process. The application of the dynamic compartment model was demonstrated in a study of an industrial fermentation process in a 600 m^3^ bubble column bioreactor. The flow model was used to evaluate the mixing performance by means of tracer simulations and was coupled with reaction kinetics to simulate concentration gradients in the process. The simulations showed that despite the presence of long mixing times and significant substrate gradients early in the process, improving the heterogeneity did not lead to overall improvements in the process. Improvements could, however, be achieved by modifying the dextrose feeding profile.

**Nomenclature**
*C*
: Concentration (kg m^−3^)
*C*_probe_
: Probe clearance (m)
*D*
: Reactor diameter (m)
*F*
: Mass flow rate (kg s^−1^)
*H_L_*
: Liquid height (m)
*k_L_a*
: Mass transfer coefficient (hr^−1^)
*P*
: Pressure (Pa)
*Q*
: Volumetric flow rate (m^3^ s^−1^)
*r*
: Specific rate (kg kg^−1^ hr^−1^)
*q*
: Volumetric rate (kg m^−3^ hr^−1^)
*T*
: Temperature (K)
*V*
: Volume (m^3^)
*v_s_*
: Superficial gas velocity (m s^−1^)
*Y*
: Mass yield coefficient (kg kg^−1^)
*ϵ*
: Gas fraction (-)
*μ*
: Specific growth rate (hr^−1^)
*ρ_p_*
: Particle density (kg m^−3^)
*ρ_l_*
: Liquid density (kg m^−3^)
*ρ_f_*
: Fluid density (kg m^−3^)
**Subscripts**
: **Description**
*m*
: Maintenance
*0*
: Initial condition
*p*
: Product
*x*
: Cell biomass
*s*
: Substrate

## Introduction

Maximizing the rate, titer, and mass yield (i.e., kilogram of product per kilogram of substrate) of fermentations are key economic considerations in industrial bioprocesses (McClure et al., 2016). Yield on substrate, such as dextrose (industrial glucose), can represent up to 65–85% of the variable cost of manufacture for commercial fermentation processes and therefore has a critical impact on economics of the overall biomanufacturing process (Sanford et al., 2016). A reduced yield may be observed in fermentation processes when operated in full-scale bioreactors compared to the laboratory-scale bioreactors, in which the processes have been developed (Enfors et al., 2001; George et al., 1998). This yield gap, which occurs on process scale-up, has been attributed to heterogeneities in process variables, such as pH, dissolved gases (O_2_ and CO_2_), and substrate concentration (Wehrs et al., 2019). Heterogeneity is present because it is technically infeasible to completely homogenize the fermentation broths of large-scale bioreactors (≫10 000 L), and an axial gradient in the hydrostatic pressure exists due to the large liquid volumes, which affects the gas transfer in the broth (Vrábel et al., 2000; Wehrs et al., 2019).

Optimal operation of fermentation processes under heterogeneous conditions is not trivial and requires deep understanding about the extent of the heterogeneity and its impact on the production organism. Mathematical modeling serves as an important tool to obtain this understanding and provide a quantitative basis for process optimization, design, and control (Gernaey et al., 2010). Modeling of large-scale fermentation processes is challenging because of the complexity involved when combining mixing, mass/heat transfer, and reactions over a wide range of time and length scales (Pigou & Morchain, 2015). The combination of numerical derivation of flows and phase interactions using computational fluid dynamics (CFD) and metabolic models has proven a powerful tool to study gradients in large-scale bioreactors (Bach, 2019; Haringa et al., 2016; Kuschel et al., 2017; Larsson et al., 1996; Morchain et al., 2014; Siebler et al., 2019). However, accurate CFD-modeling of gas–liquid interactions and viscosities of non-Newtonian fluids is challenging, and simulations coupled with metabolic models are computationally demanding (Haringa et al., 2016; Nauha et al., 2018). This makes simpler flow models with lower associated computation times, such as compartment models, attractive for modeling of large-scale bioreactors. Indeed, CFD has lately been applied extensively to develop compartment models of bioreactors (Bezzo & Macchietto, 2004; Delafosse et al., 2014; Nauha et al., 2018; Nørregaard et al., 2019; Tajsoleiman et al., 2019). However, such models cannot be effectively applied for long-lasting processes with dynamic changes to the flow field, volume, and fermentation broth rheology, which is the situation of the widely used fed-batch process. Jourdan et al. propose a solution that involves a set of discrete steady-state CFD simulations accompanied by an algorithm for automatic conversion into compartment models (Jourdan et al., 2019). A solution with such a dynamic compartment model has been presented in a study of heterogeneity in an industrial aerobic fed-batch fermentation process with *Saccharomyces cerevisiae* (Nadal-Rey et al., 2021). Despite providing detailed insights with a purely computational methodology, the approach is still time-consuming if the process is to be sufficiently resolved in time and potentially suffers from inaccuracies when unvalidated CFD models of such complexity are applied.

Detailed measurements from large-scale processes can be obtained by flow-following sensor devices, which have recently been demonstrated in a study that examines hydrodynamics in a 2 077 m^3^ activated sludge basin at a wastewater treatment plant (Reinecke & Hampel, 2017). Such sensor devices provide a detailed representation of the spatial distribution of variables with simple experimental procedures because the sensor devices autonomously store or/and transmit data while they are carried along with the agitation or convection-induced fluid motion. Reinecke et al. have shown that axial velocity fields can be obtained from the measurements of hydrostatic pressure collected by the sensor devices (Reinecke et al., 2012). These axial velocity fields can be used to develop simple flow models in the form of compartment models, which has been previously demonstrated in a study of a pilot-scale stirred vessel (Bisgaard et al., 2021b). The developed compartment model approach includes automatic compartmentalization and determination and the assignment of inter-compartment flow rates between the compartments.

This paper presents a methodology for developing compartment models based on data from flow-following sensor devices, which is tailored for application in large-scale fed-batch fermentations. The methodology is based on the compartment model approach presented in a previous study (Bisgaard et al., 2021b), which is expanded to include a dynamic update of volume and inter-compartmental flow rates in time. The development and application of the model are demonstrated in a study of a fermentation process that utilizes an *Escherichia coli* biocatalyst to produce 1,3-propanediol in a large-scale bubble column bioreactor. The goal is to assess and minimize risks associated with making physical and functional modifications to the fermenter used in a commercial manufacturing process. In the absence of data, certain changes can have significant negative consequences on profitability, product quality, and other important factors related to 1,3-propanediol production. For the assessment, the developed compartment model is coupled with a kinetic model to investigate the process in terms of mixing performance and concentration gradients.

## Materials and Methods

### Bioreactor and Fermentation

**Fig. 1:**
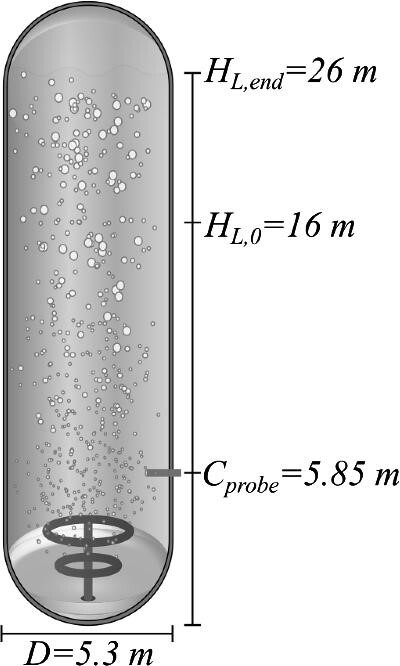
Illustration of the bubble column bioreactor with annotations of the dimensions that are relevant for the modeling.

**Fig. 2:**
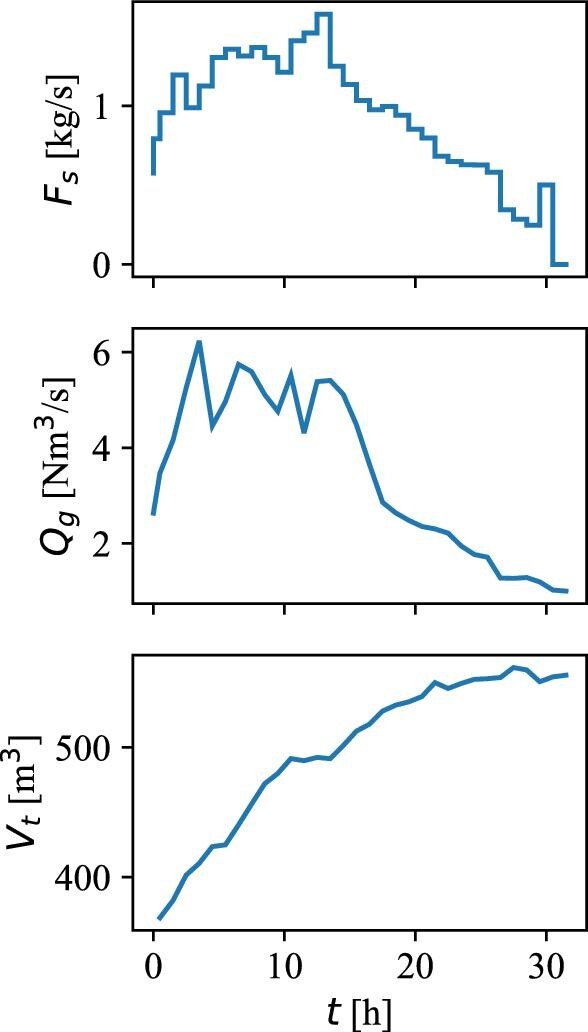
Profiles of the feed rate *F*_s_ (top), gas flow rate *Q*_g_ at *T* = 298.15 K and *P* = 101 325 Pa (middle), and total volume *V*_t_ (bottom) during the fermentation process.

The experiment was performed in a bubble column bioreactor (DuPont Tate & Lyle Bio Products Company, TN, USA) with a diameter (*T*) of 5.3 m and a total volume (*V*) of 600 m^3^ (Fig. 1) (Crater et al., 2017). The examined bioprocess utilizes a recombinant *E. coli* K-12 biocatalyst to convert corn syrup (>95% β-D-glucose, also known as dextrose) to 1,3-propanediol (PDO) (Nakamura & Whited, 2003). During the 32-hr fermentation process, which was operated as a fed-batch (Guske & Miller, 2009), the gassed liquid level increased from an initial level of 16 m (*H_L_*_,0_) to a final level of 26 m (*H_L_*_, end_) from dextrose substrate addition. The initial volume in the bioreactor consisted of an M9 minimal media (Antoniewicz et al., 2007) with 10% inoculum from a seed fermenter. The dextrose substrate and media were added to the liquid surface with flow rates according to the profile in Fig. 2 (top), while air was added through two inlet spargers at the bottom of the bioreactor with the flow rates according to Fig. 2 (center). The increase in gassed bioreactor volume during the fermentation is shown in Fig. 2 (bottom).

### Description of the Biocatalyst Used for Production of 1,3-Propanediol

The dextrose-based fed-batch fermentation process utilized a genetically modified derivative of *E*. *coli* K-12 (Genbank Accession number U00096.3) that lacked λ-DNA sequence and F plasmid (F^−^). Biosynthesis of 1,3-propanediol is driven by a predominantly heterologous carbon pathway that diverts carbon from dihydroxyacetone phosphate to form 1,3-propanediol. As reviewed by Nakamura and Whited (2003), modifications to the host strain include the introduction of glycerol 3-phosphate dehydrogenase (DAR1) and glycerol 3-phosphate phosphatase (GPP2) genes obtained from *S*. *cerevisiae* to provide glycerol. Glycerol dehydratase (dhaB1, dhaB2, dhaB3) and its reactivating factors (dhaBX, orfX) obtained from *Klebsiella pneumoniae* enable the conversion of glycerol to 3-hydroxypropionaldehyde, while the synthetic oxidoreductase, originally endogenous to *E. coli* (yqhD), completes the pathway.

Non-productive or yield-reducing reactions, including acetate production, were attenuated or eliminated through gene deletions, and oxygen-regulated gene expression (arcA) has been disrupted (Cervin et al., 2010; Nakamura & Whited, 2003). Glycerol is prevented from re-entering central carbon metabolism by deletion of the genes encoding glycerol kinase (glpK) and glycerol dehydrogenase (gldA). The strain features elimination of D-glucose transport by the phosphotransferase system (PTS) and downregulation of glyceraldehyde 3-phosphate dehydrogenase (*gapA*). The PTS system is replaced with a synthetic system comprising galactose permease (*galP*) and glucokinase (*glk*), genes endogenous to *E. coli*. The galP and glk system has been observed to significantly increase the rate of substrate uptake and improve the yield of 1,3-propanediol from glucose (Antoniewicz et al., 2007; Nakamura & Whited, 2003).

### Mixing Time of a Chemical Tracer

The 95% mixing time (*t_m95_*) was determined experimentally using the chemical tracer method of Hadjiev et al. (2006) and reported previously by Guske & Miller (2009). The mixing time was measured using a 100 L pulse injection of 2 M NaOH that was added to the top surface of the bubble column bioreactor at an addition rate of 10.5 L/s. Mixing time measurements were initiated at the start of the NaOH pulse and followed by measurements of the pH value sampled at 1 Hz using an *in situ* Mettler-Toledo pH sensor (Metter-Toledo model InPro3100). The pH sensor was located within 10 cm of the bioreactor wall and 5.85 m above the bottom drain valve of the reactor. Simulated bioreactor run conditions at a fermentation elapsed time of 15 hr for the 1,3-propanediol bioprocess were used to assess mixing time following the pulse of 2 M NaOH. The reactor was filled with 490 m^3^ of reverse osmosis-grade water and a total of 10 separate measurements were performed using the following conditions: an airflow of 4.75 Nm^3^/s, a bioreactor back pressure of 21 kPa, and liquid temperature 33°C. The mean for the 10 independent measurements is reported as the ‘‘measured mixing time at 15 hours’’ (Fig. 5). The standard error for measurements was found to be within the error range (3–5%) previously reported by Hadjiev et al. (2006).

### Measurement of Variables

Samples from the bioreactor were taken during the fed-batch fermentation and analyzed for biomass concentration, fermentation analytes by high-performance liquid chromatography (HPLC), and glucose by enzyme-linked amperometric electrode using a Yellow Springs Instruments (YSI, Yellow Springs, OH, USA) model 2950D biochemistry analyzer. Biomass concentration was determined by measuring the optical density of the broth sample at 550 nm, assuming 3.0 g/L/OD550 cell dry weight and 25.3 g/C-mol for molecular weight of dry biomass (Antoniewicz et al., 2007). Online process data collected from the bioreactor included dissolved oxygen (DO) using *in situ* probes installed in the bioreactor at the vessel wall exactly 5.85 m from the bottom drain valve. Digital optical DO sensors (Mettler-Toledo, model InPro6860i, Columbus, OH, USA) and pH sensors (Metter-Toledo model InPro3100) were sampled at a measurement frequency of 1 Hz.

For HPLC analysis, broth samples were centrifuged at 10 000 g for 10 min, and the supernatant was filtered into a 2 mL autosampler vial using a 25 mm 0.45 µm Agilent Captiva layered syringe filter (glass microfiber pre-filter, PTFE membrane; part number #5190-5129). A modification of the method by Zaky et al. (2017) was utilized for the analysis of samples using an Agilent 1260 HPLC with refractive index (RI) and ultraviolet (UV) detector for carbohydrates/alcohols and organic acids, respectively. This isocratic method was modified for simultaneous separation of carbohydrates, alcohols, and organic acids that were present in the fermentation broth samples. This system consisted of two—Hi-Plex H, 300 mm × 7.7 mm Organic Analysis Column (Agilent Part Number PL1170-6830, Santa Clara, CA, USA) analytical columns that were connected in series. A guard column was installed before the analytical column and consisted of a Cation-H Cartridge 30 × 4.6 mm Guard Column (Bio-Rad Part Number 125-0129, Hercules, CA, USA). Instrument conditions were as follows: mobile phase consisting of 10.0 mM sulfuric acid (Sigma Catalog # S1526) in HPLC-grade water, isocratic pump flow rate of 0.60 mL/min, column temperature of 70°C, injection volume of 10 μL, UV wavelength of 210 nm, RI temperature of 55°C, and a run time of 55 min.

### Flow-Following Sensor Devices

The bubble column bioreactor was examined using flow-following sensor devices (Freesense ApS, Denmark) (Freesense ApS, 2022), which were introduced to the bioreactor prior to steam sterilization. The sensor devices measuring 55 mm in diameter had their density adjusted based on initial calculations of the fluid density, motivated by results from Middleton (1979), which showed that large particles behave more like the liquid when the density of the particle resembles the density of the gas–liquid dispersion. Sensor devices with the following densities were used in the experiment: two sensor devices with a density (*ρ_p_*) of 850 kg/m3, three sensor devices with a density of 900 kg/m^3^, and three sensor devices with a density of 950 kg/m^3^. The sensor devices were configured to collect and store measurements of the pressure and temperature at a sampling rate of 1 Hz during the entire duration of the fermentation process.

#### Processing of sensor device data

The axial position (*z(t)*) and axial velocity (*v_z_(t)*) were derived from measurements of hydrostatic pressure, using Pascal's law, presented in *Equation 1*.

(1) \begin{equation*}z\ \left( t \right) = \frac{{{P}_{{\rm{max}}} - P\left( t \right)}}{{{\rho }_f \cdot g}}.\end{equation*}

In the equation, *z(t)* denotes the axial position at time *t, P(t)* is the measured pressure at time *t, P_max_* is the maximum measured pressure (which occurs at the bottom of the reactor), *ρ_f_* is the fluid density, and *g* is the gravitational acceleration. Further details on the derivation of the axial position and the axial velocity are presented in a previous study (Bisgaard et al., 2021a). Three contributions to variations in the measured pressure difference (*P_max_–P(t)*) had to be considered before the conversion of pressure to the axial position. Firstly, contributions from variations in head space pressure due to aeration, which result in a shift in *P_max_*. Secondly, contributions from volume increase due to substrate addition, which result in an increase in *P_max_*, and thirdly, contributions from variations in the volume resulting from changes in the fluid density. The contributions to the pressure difference resulting from the head-space pressure and the volume changes were compensated by subtraction of a baseline obtained from a rolling-maximum filter with a 1-hr window size, which was further smoothed by a rolling-averaging filter. The baseline represents the dynamic changes in the pressure at the bottom of the reactor over the duration of the fermentation process. This approach assumes that the sensor devices reach the top and bottom of the bioreactor in this 1-hr timeframe and that negligible changes to the liquid height occur within this 1-hr period, which is both reasonable assumptions. Changes to the fluid density are mainly attributed to changes in the gas hold-up, which was estimated for a heterogeneous flow regime by the correlation from van't Riet & Tramper (1991) in *Equation 2*.

(2) \begin{equation*}\epsilon \ = \frac{{{v}_s}}{{0.25 + 0.45{{\left( {g\ {v}_s\ D} \right)}}^{\frac{1}{3}}}}.\end{equation*}

The fluid density of the gas–liquid dispersion was then calculated from *ρ_f_* = (1–*ε*)*ρ_l_*−*ερ_g_* (Hofmeester, 1988), with *ρ_l_* = 1030 kg/m^3^. For further details on the estimated fluid density, refer to ‘‘Supplementary Materials’’. The sensor devices with densities of 950 kg/m^3^ were found to be closest to the mean fluid density for the largest part of the process. Hence, data from these sensor devices were used to generate the compartment model. Regardless, it was found that the sensitivity of the mixing time predictions from compartment models developed based on data from sensor devices with different densities was low (refer to ‘‘Supplementary Materials’’). Instantaneous pressure measurements from the sensor devices were used to correct the superficial gas velocities and calculate the instantaneous gas hold-up and fluid density. Ultimately, the instantaneous fluid density was used in the calculation of the axial position and axial velocities by Pascal's law (*Equation 1*).

## Modeling

### Dynamic Compartment Model

**Fig. 3:**
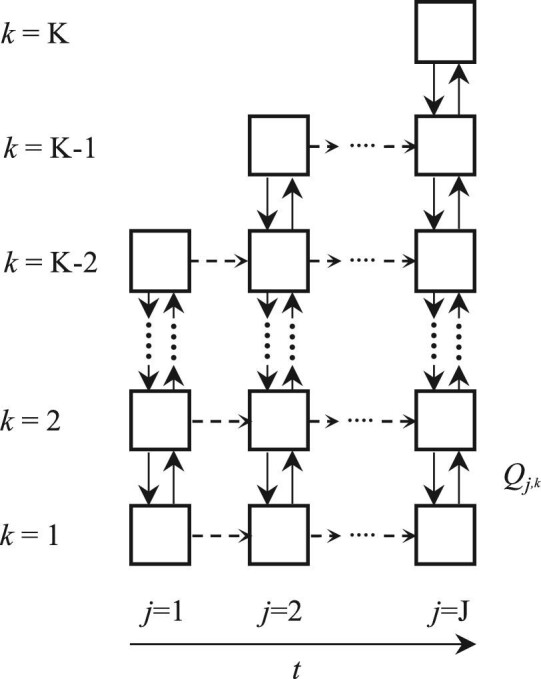
Schematic representation of the dynamic compartment model with K compartments at the final volume. Mass is exchanged between the ideally mixed compartments with the flow rate *Q*, denoted by the bidirectional vertical arrows. The total volume and the flow rates are updated in time in discrete model update steps (*j* = 1,…, *J*). The dashed horizontal arrows denote transfer of mass between model update steps.

The dynamic compartment model is based on axial compartments (*k* = 1,…, *K*) with volumes (*V_k_*), which are interlinked by bidirectional axial flows (*Q_k_*). The derivation of the volumes and the flow rates of the axial flows between the compartments are described in detail in a previous paper (Bisgaard et al., 2021b). In contrast to the preceding study, in which steady experimental conditions were examined, the total volume and the flow rates between compartments are functions of time. As the sensor devices provide Lagrangian-type measurements, i.e., measurements that are functions of time and instantaneous position, the measurements are discretized in both space (compartments) and time by averaging of the measured variables. Discretization in time is performed by introducing compartment model update steps, denoted by *j =* 1*,…, J*. From one compartment model update step to another, the total reactor volume of the model is updated by modifying the liquid height, using *Equation 1* with the pressure difference between the maximum and minimum pressure encountered during the update step. Furthermore, all inter-compartment flow rates are updated according to the changes in axial velocity at each update step. The compartment volumes and inter-compartment flow rates are scaled with the average liquid fraction (1–*ε*) to account for the dynamic gas-holdup, which affects the effective compartment interface area and compartment volume and therefore the flow rates at the compartment interfaces and the concentrations in the compartments. Within each update step, the total volume and the flow rates between compartments are assumed constant. A schematic representation of the dynamic compartment model is shown in Fig. 3.

The horizontal dashed lines represent the transfer of mass between the update steps, which are addressed in ‘‘Fermentation simulations’’ Section. Pressures and temperatures measured by the sensor devices are also assigned to compartments, which are ultimately used for the calculation of the gas mass transfer coefficient *k_L_a*. An update frequency of 1 hr and an initial compartment height of 0.5 m were found to be appropriate based on a requirement of a minimum of 30 measurements of the axial velocity at any compartment interface in the model.

**Fig. 4:**
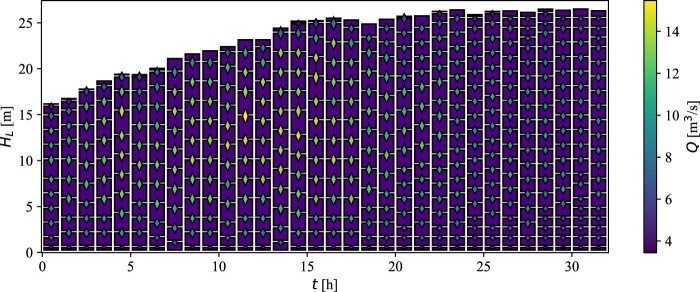
Automatic zoning of the volumes in the compartment model. The compartments are presented as purple squares, separated by black lines at the compartment interfaces. The diamonds at the interfaces represent the flow rates according to the color bar. At high flow rates, more of the initial compartments are merged to larger compartments, e.g., at *t* ≈ 15 hr, while fewer of the initial compartments are combined at the low flow rates at the end of the process, e.g., *t* ≈ 30 hr, especially toward the top and bottom.

The initial compartments were combined into the zones depicted in Fig. 4, based on the automatic zoning procedure presented in a previously published paper (Bisgaard et al., 2021b). The auto-zoning model parameter, the local critical residence time (*τ_crit_*), was modified from *τ_crit_* = 0.95 s found in Bisgaard et al. (2021b) to *τ_crit_* = 1.5 s to obtain a better fit with experimental mixing time data (presented in ‘‘Mixing’’ Section). The critical local residence time is a threshold parameter that decides on the volume of compartments for which the assumption of ideal mixing is appropriate. The local residence time, which is defined by the volume to flow rate ratio in the compartment, must not exceed the critical local residence time for the assumption of perfect mixing to be appropriate.

### Fermentation Modeling

The fermentation process was modeled with differential *Equations 3**–**6*. The concentration of variable *i* is denoted *C_i_*, while *r_i_* denotes the specific rate of change of variable *i*. Biomass, substrate, product, and oxygen are denoted by subscripts *x, s, p*, and *o*, respectively. The index *j* refers to the compartment model update step, which implies that properties with index *j* are updated at discrete time steps.

(3) \begin{equation*}\frac{{d{C}_x}}{{dt}} = \ \mu {C}_x,\end{equation*}

(4) \begin{equation*}\frac{{d{C}_s}}{{dt}} = \ - {r}_s{C}_x + {F}_{s,j,k\ = \ K},\end{equation*}

(5) \begin{equation*}\frac{{d{C}_p}}{{dt}} = {r}_p\ {C}_x,\end{equation*}

(6) \begin{equation*}\frac{{d{C}_o}}{{dt}} = {k}_L\ {a}_{\left( {j,\ k} \right)}\left( {C_{o,j}^* - {C}_o} \right) - {r}_o{C}_x.\end{equation*}

The feed addition was modeled by the mass flow rate term *F_s_,(_j, k__=__K__)_*, which adds substrate to the top compartment (*k* = K) according to the feed rate profile in Fig. 2 (top). The feed rate is updated for each compartment update step (*j*) but is constant throughout the update step and does not induce changes to the volume during the step.

The mass transfer coefficient, *k_L_a(_j, k)_*, was modeled with a simple linear relationship with the superficial gas velocity *k_L_a* = 0.288 *v_s_* (Guske & Miller, 2009). The superficial gas velocity (*v_s_*) was calculated based on the airflow in Fig. 2 (middle), which was corrected by the average pressure and temperature measured by the sensor device in the corresponding compartments. The saturated molar oxygen concentration (C*_o_,_j_) was estimated by multiplying the average total pressure in the compartments by the mole fraction of oxygen in the air (*x*_O2_ = 0.2095), followed by the application of Henry's law with the assumption that the way oxygen solubilizes in the fermentation broth is similar to pure water. This is a rough assumption because the solubility of oxygen in the culture medium containing various salts differs from the solubility in pure water and may keep changing throughout the fermentation. A temperature-compensated Henry's coefficient for oxygen in water of *H^cp^*(*T* = 306 K) = 0.0015 mol/L/atm was used for the calculation (Sander, 2015).

#### Specific rates

The biomass-specific growth rate (µ) was modeled using Monod kinetics with product inhibition (*Equation 7*) as proposed in Ross et al. (1994). Here, *K_p_* is the critical product concentration for which *µ* = 0. Negative values for the growth rate are allowed for *C_p_* > *K_p_*, corresponding to cell death. Because the strain is incapable of regulating its metabolism to anaerobic growth conditions (‘‘Description of the Biocatalyst Used for Production of 1,3-Propanediol’’ Section), a term *C_o_*/(*C_o_* + *K_o_*) is added to account for situations where oxygen becomes growth limiting.

(7) \begin{equation*}\mu \ = {\mu }_{{\rm{max}}}\ \cdot \left( {\frac{{{C}_s}}{{{C}_s + {K}_s}}} \right) \cdot \left( {\frac{{{C}_o}}{{{C}_o + {K}_o}}} \right) \cdot \left( {1 - \frac{{{C}_p}}{{{K}_p}}} \right).\end{equation*}

The production of PDO is modeled by the Luedeking–Piret model (Luedeking & Piret, 1959), which states a linear relationship between specific product formation rate (*r_p_*) and both biomass concentration and specific growth rate (*Equation 8*). The PDO precursor, glycerol, is assumed not to be accumulating in the broth because the rate-limiting step is expected to be earlier in the pathway. Modeling of glycerol is therefore omitted.

(8) \begin{equation*}{r}_p = {Y}_{px}\ \mu + {r}_{x,p}.\end{equation*}

Specific uptake rates of substrate (*rs*, *Equation 9*) and oxygen (*r_o_*, *Equation 10*) are modeled by division of the specific rates with corresponding stoichiometric yield coefficients *Y_sx_, Y_ps_*, and *Y_so_*. Terms for substrate and oxygen uptake for biomass maintenance were included as the specific rates *r_m_,_s_* and *r_m_,_o_*.

(9) \begin{equation*}{r}_s = \frac{\mu }{{{Y}_{xs}}}\ + \frac{{{r}_p}}{{{Y}_{ps}}} + {r}_{m,s},\end{equation*}

(10) \begin{equation*}{r}_o = \frac{{{r}_s}}{{{Y}_{so}}}\ + {r}_{m,o}.\end{equation*}

The genetic modifications of the *E. coli* K-12 strain are expected to have an impact on the model parameters when compared to the model parameters of the wild-type *E. coli*, e.g., the parameters presented in the paper by Xu et al. (1999). The model parameters were therefore fitted by minimizing the sum of squared errors (SSE) between the simulated and the measured values of biomass, substrate, and oxygen, using the Nelder–Mead optimization algorithm from the Python library, Scipy. For the SSE between simulated and measured biomass, product, and substrate concentration, the average concentration of compartments across the liquid height was used, while for the DO, the oxygen concentration at the compartment closest to the DO probe location (*C_probe_* = 5.85 m) was used. The fitted values were not constrained by a stoichiometric equation. A table with the fitted parameters and the variables used in the modeling are presented in ‘‘Supplementary Materials’’.

### Simulations

#### Mixing time simulations

The tracer concentration transients were simulated at each compartment model update step by initializing an arbitrary tracer concentration in the top compartment volume and numerically solving the ordinary differential equations using the LSODA solver implementation from Python's Scipy library. The time to reach 95% homogeneity (*t_m95_*) was determined from the logarithmic root mean square variance of the normalized responses in all the modeled compartments. The homogenized concentration (*C_inf_*) was taken as the concentration of the tracer after 10 min. The process was repeated for a set of different feeding points.

#### Fermentation simulations

The fermentation process was simulated by solving the model states using an ordinary differential equation solver (LSODA algorithm, Python's Scipy library). The compartment volumes at specific liquid heights are subject to change between compartment model update steps (Fig. 4). Therefore, the final concentrations of the simulated variables at each update step had to be processed before being used as initial conditions for the next compartment update step. This was done by first converting concentrations to masses by multiplication with the corresponding compartment volumes of the current update step. Second, the masses were diluted into temporary compartments resembling the compartments of the current step, but with volumes that have been scaled with the ratio between the total volume of the next update step and the current update step (*V_j+1_*/*V_j_*). Finally, the masses of the simulated variables in the following update step were calculated based on the volume ratio between the temporary update step and the following step, e.g., if a compartment of the temporary update step is divided in two by an interface in the middle at the following update step, the mass from the current step is divided fifty–fifty into the compartments of the following update step.

## Results and Discussion

### Mixing

**Fig. 5:**
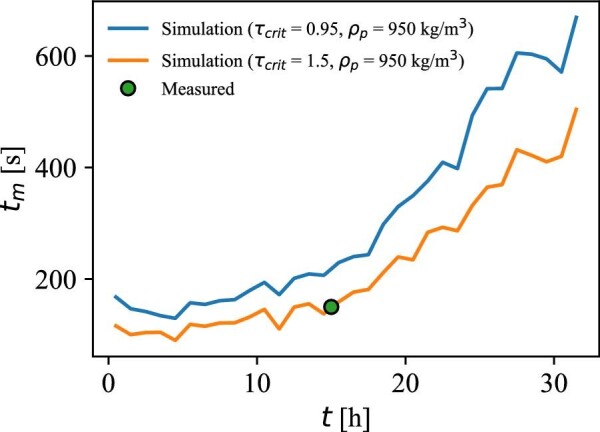
The simulated mixing time with *τ*_crit_ = 0.95 s (blue line) and *τ*_crit_ = 1.5 s (orange line), and the measured mixing time at *t* = 15 hr (green circle).

The results from the mixing time simulations with two different values of the model parameter *τ_crit_* (*τ_crit_* = 0.95 s and *τ_crit_* = 1.5 s), together with the measured mixing time at *t* = 15 hr, is presented in Fig. 5.

As mentioned previously, a value for the parameter *τ_crit_* of 1.5 s was used instead of the previously fitted value of *τ_crit_* = 0.95 s (Bisgaard et al., 2021b), because of the better fit with experimental data. Using a value of *τ_crit_* = 0.95 s resulted in a relative error of 45% at *t* = 15 hr. Potential sources of errors affecting this include inaccurate estimation of the fluid density, the assumption about perfect radial mixing from an exclusively axial compartment model, and overestimation of the bottom volume because it is considered to be cylindric in the model, whereas it is semi-elliptical in reality. Inaccuracies in the estimated fluid density affect the calculation of the axial position (and velocity) and hence the volume of the compartments and the derived flow rates between them. More details about how inaccuracies in the fluid density affect the calculated position and the sensor device buoyancy have been addressed in previous work (Bisgaard et al., 2020). The buoyancy of the sensor devices may be affected if a gradient in the fluid density exists over the liquid height. However, an analysis presented in ‘‘Supplementary Materials’’ indicates that the buoyancy of the sensor devices has little impact on the determined mixing times. Due to the liquid height to diameter ratio, which increases from *H_L_*/*T* = 3 initially to *H_L_/T* = 5 toward the end of the process, it is expected that the bottleneck in the mixing process occurs in the axial direction, and hence the assumption of perfect radial mixing is acceptable.

The simulated 95% mixing time is around 100 s in the beginning of the process and then starts to increase rapidly after 15 hr into the fermentation process, where it from this point continuously increases up to almost five times the initial mixing time. Because the gas flow is responsible for the mixing in bubble columns, the simulated mixing time profile agrees well with what is expected from the aeration profile in Fig. 2 and the derived axial flow rates in Fig. 4. Furthermore, the volume of the bioreactor increases continuously, which further contributes to the rise in the mixing time.

**Fig. 6:**
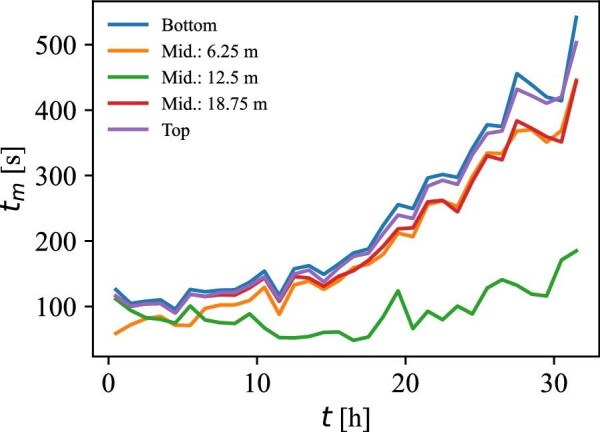
The simulated mixing time using various feeding points (compartments) in the reactor. Feeding from a point in the middle of the liquid height drastically reduces the 95 % mixing time.

Simulations with various feeding points were performed to investigate whether a certain feeding location led to improvements in the mixing time. The mixing time results from simulations where the tracer was initialized at the bottom, in three compartments in the middle, and at the top are compared in Fig. 6.

The top and bottom feeding results in significantly longer 95% mixing times since the axial flow rates are significantly lower in these zones compared to the compartments in the middle of the bioreactor and because the distance that the tracer travels is twice as long compared to the feeding points in the middle of the bioreactor. The mixing time obtained when feeding at the 12.5 m feeding point is almost unchanged over the process duration. This is because the feeding height of 12.5 m is initially closer to the liquid surface, where the axial mixing is poor. Due to the volume increase over the course of the process, the feeding height of 12.5 m advances toward the middle of the liquid height. Consequently, it can be deduced that the optimal feeding scenario of a single active sterile feed injection point would be around 12.5 m from the bottom.

### Fermentation

**Fig. 7:**
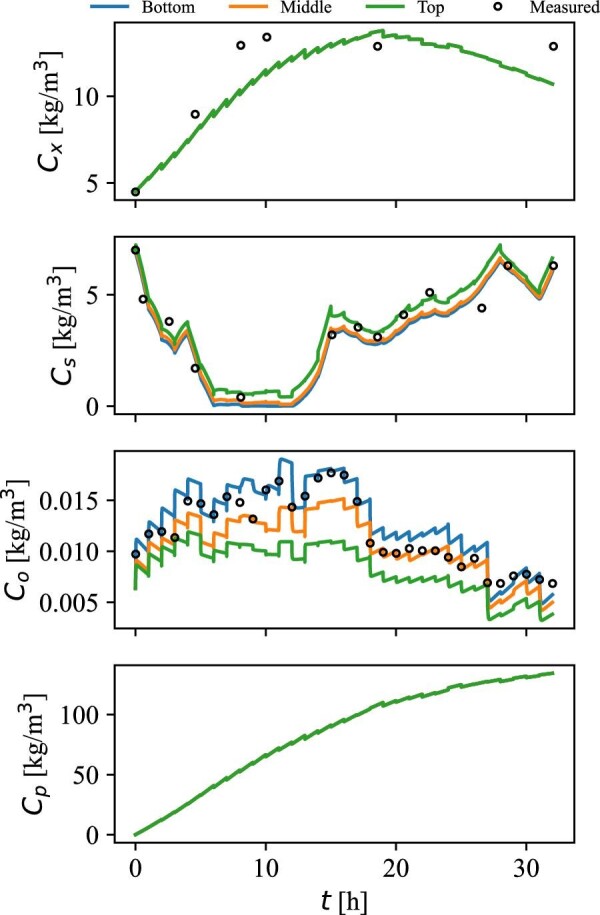
Simulated (lines) and measured (circles) concentrations of biomass (*C*_x_), substrate (*C*_s_), dissolved oxygen (*C*_o_), and PDO (*C*_p_) in the top, middle, and bottom of the bioreactor.

The simulated concentrations of biomass, substrate, DO, and product in the bottom, middle, and top of the bioreactor are presented in Fig. 7, together with the corresponding measurements of the variables.

**Fig. 8:**
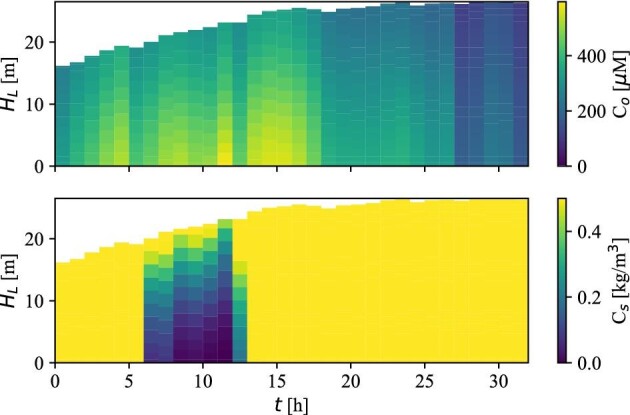
Dynamic compartment model heatmap showing the dissolved oxygen concentration (top) and substrate concentration (bottom). The displayed substrate concentrations have been capped at 0.5 kg/m^3^ to emphasize the periods with very low substrate concentrations (≈6–13 hr).

The simulated concentrations of substrate and DO agree well with the measured values throughout the process. However, with the optimal parameter fit, the simulated biomass concentration is significantly lower than the measured concentration. This may be explained by the fact that the simulated biomass consists of active cells only, while measurements of dry cell weight based on optical density represent both viable and non-viable cells. The biomass initially presents in the medium was produced during the seed fermentation prior to this process. PDO reaches a concentration of 135 kg/m^3^, which is in agreement with the value reported by Nakamura and Whited Nakamura & Whited (2003). No spatial variations are observed in the biomass and product concentration, which is expected due to the lower rates and no continuous additions. However, gradients are observed in both substrate and DO concentration, which are visualized in Fig. 8.

The top heatmap in Fig. 8 reveals that despite the presence of oxygen gradients, plenty of oxygen is present throughout the process; particularly in the important part early in the process, where product inhibition is low, and growth and production rates are high. In the case of substrate (Fig. 8, bottom), a period with low substrate concentrations between *t* = 6 hr and *t* = 13 hr is present. The low concentrations are especially prevalent toward the bottom of the reactor, where the concentration approaches zero.

**Fig. 9:**
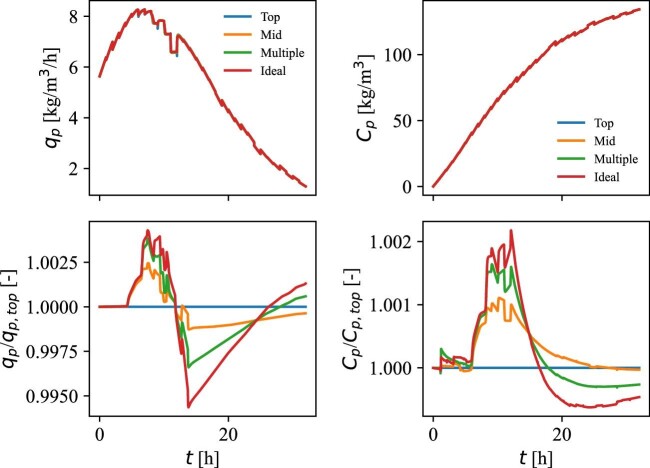
Productivity, *q*_p_ (top, left), product concentration, *C*_p_ (top, right), productivity ratio between the current situation and the three additional examined cases (bottom, left), and product concentration ratio between the current situation and the three additional examined cases (bottom, left).

Whether this period of low substrate concentration has an impact on the process is examined by comparing the productivity between four cases. The first case is the present situation with substrate feeding to the liquid surface, the second case includes substrate feeding at 12.5 m from the bottom, the third case includes a situation where one-third of the feed is maintained at the top, while two-thirds of the feed are added at another feeding point 5 m from the bottom, and finally, the last case includes a situation with ideal mixing. In practice, the ideal mixing case was simulated by simply raising the flow rates in the compartment model to extremely high values. The simulation results of productivity (*q_p_*) and product concentration (*C_p_*) for the four cases are presented in Fig. 9, together with the improvement/worsening in the different cases compared to the current feeding strategy using the top feeding point.

The productivities in the four cases are shown in the top left of Fig. 9. Initially, the productivity increases from less than 6 kg/m^3^/hr to approx. 8 kg/m^3^/hr due to the favorable growth conditions and an increase in the total biomass. From this maximum, the growth-associated productivity decreases linearly with the inhibition of the growth rate by the PDO to reach final productivity of 1 kg/m^3^/hr. The values obtained here are therefore slightly higher than the productivity of 3.5 kg/m^3^/hr reported in the literature (Nakamura & Whited, 2003). Despite the observed improvement in mixing performance when feeding to the middle of the liquid height (‘‘Mixing’’ Section), only insignificant improvements to productivity and product concentration in the period of 6–13 hr were observed (Fig. 9, bottom left and right). The case with multiple feeding points showed to be slightly better in the same period, while the case of ideal mixing has the greatest improvements in the period. However, the improvements in any of the cases are << 1% and only result in insignificant improvements in the product concentration (Fig. 9, bottom right). Furthermore, after the period of improved production (6–13 hr), a period of lower production compared to the current feeding strategy is present. This is a result of a higher product concentration compared to biomass in the three examined cases compared to the current situation, which leads to an increased product inhibition. Ultimately, the examined cases result in less product compared to the current situation if the process duration is maintained, but with negligible differences. This underlines the robustness of the process due to the genetic modifications of the production strain, which allow the cell population to grow at high respiration and substrate uptake rates without excretion of acetate to the medium. Therefore, the average substrate concentration can be maintained at a relatively high level throughout the process, avoiding problems with mixing limitations in the process.

**Fig. 10:**
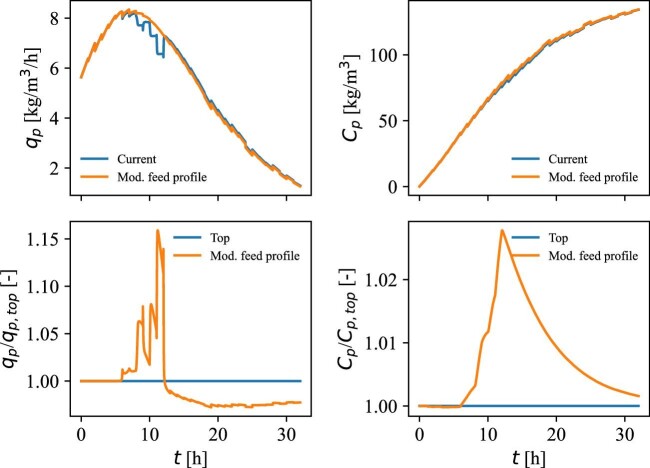
Productivity, *q*_p_ (top, left), product concentration, *C*_p_ (top, right), productivity ratio between the current situation and a case with a modified feeding profile (bottom, left), and product concentration ratio between the current situation and the examined case (bottom, left).

As the substrate is completely consumed in the period from 6 to 13 hr and plenty of substrate is available later (>13 hr), an adjustment to the feed rate profile of the process may lead to improvements. The results from a case where the feed rate was increased with 0.07 kg/s for a 7-hr period between 6 and 13 hr and then reduced the same amount for a period between 23 and 30 hr are presented in Fig. 10.

In this case, the productivity is improved with up to 15% in the period from 6 to 13 hr, which results in a 2.5% higher product concentration 13 hr into the process. However, due to product inhibition, this advantage is reduced over time, resulting in just minor improvements after 32 hr.

A more general optimization could aim to optimize the productivity while minimizing the total substrate addition. However, batch-to-batch variations occur in the uptake rates, and it may not be desirable to push the feed addition to the limit. The model presented in this paper is rather simplistic, while further improvements to kinetic model would include the modeling of CO_2_, which has an inhibitory effect on growth and production, and modeling the PDO precursor glycerol, which is known to be present at significant concentrations when the broth is transferred from the seed reactor to the production reactor. The model assumes instant adaptation of the cells to the reactant concentrations with no record of previous conditions. In reality, the cells may respond negatively to exposure to fluctuating conditions, e.g., between the conditions with plenty of substrate in the top of the reactor and substrate-depleted zones in the bottom of the reactor. Therefore, the effect of the gradients in the process may be underestimated.

## Conclusion

A data-based compartment model approach was expanded to a dynamic version, which enables modeling of the extensively used fed-batch process. The dynamic model introduces a set of discrete compartment model update steps, for which the total volume and inter-compartment flow rates are updated in time. With an adjustment to the model parameter *τ_crit_*, the dynamic compartment model predicted the flow dynamics and mixing performance during the entire process. Based on mixing times obtained from simulations with various tracer injection points, it was concluded that addition to the middle of the liquid height was optimal with respect to homogenization of the tracer. The dynamic compartment model was then coupled with kinetic models for growth, production, and consumption to perform simulations of concentrations of biomass, substrate, product, and DO over the course of the fed-batch fermentation. The simulations revealed that relatively long mixing times (*t_m_* ≈ 125 s) and critically low substrate concentrations were present at the bottom of the reactor between 6 and 13 hr into the process. However, model predictions indicated that improving the homogeneity during this period did not result in improvements to the process. Reallocating dextrose from later in the process to this period increased the productivity in the period with up to 15%, which resulted in a final concentration that was slightly higher than the base case. The dynamic compartment model provides a simple model framework for performing this type of simulation with a low computational demand. This makes it highly suitable for solving optimization problems, such as fitting of model parameters or optimization of feeding locations and/or rates.

## Supplementary Material

kuac021_Supplemental_File
