# Supplementary material for: Data-based dynamic compartment model: Modeling of E. coli fed-batch fermentation in a 600 m3 bubble column
Source: J Ind Microbiol Biotechnol. 2022 Sep 30;49(5):kuac021. doi: 10.1093/jimb/kuac021 (PMC9559308; doi:10.1093/jimb/kuac021)
Supplement: kuac021_Supplemental_File [file kuac021_supplemental_file.docx]

**Supplementary material**

*Density of gas-liquid dispersion*

The fluid densities were calculated based on the superficial gas velocities, which have been corrected by the effect of hydrostatic pressures (*P*) and temperature (*T*) on the gas volumes over the liquid height, using the ideal gas law *V_2_* = *V_1_*(*T_2_*/*T_1_*)(*P_1_*/*P_2_*). The temperature was found from the sensor device measurements to be homogeneous across the volume. The range is shown in Figure 1, with the line representing the mean fluid density. The fluid density decreases towards the top because the superficial gas velocity increases as a result of gas-volume expansion from a reduction in the hydrostatic pressure.


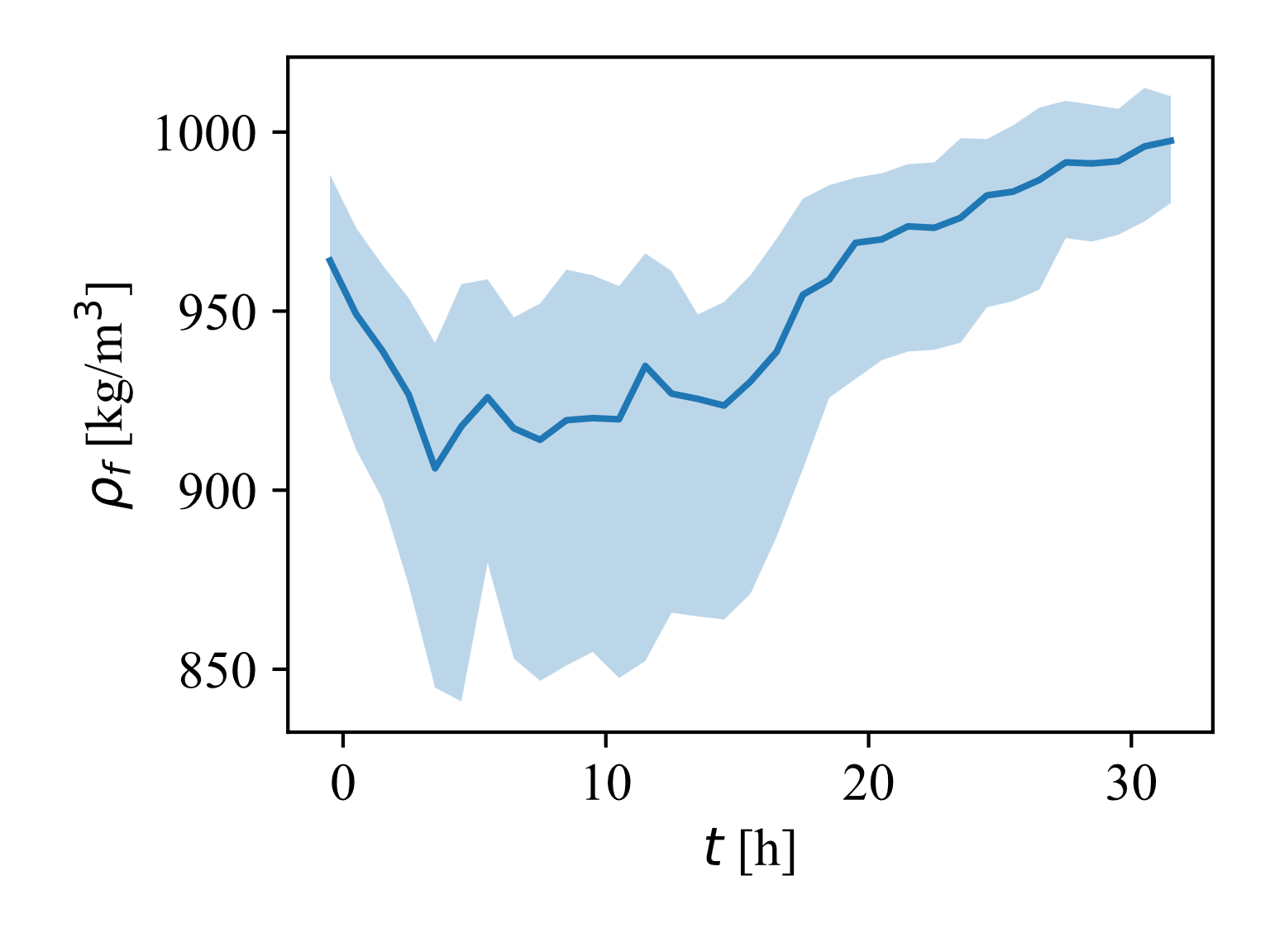


Figure 1. Estimated fluid density (ρ_f_) of the gas-liquid dispersion over the process duration. The area represents the minimum and maximum fluid density observed during 1-hour periods, while the line represents the average fluid density during these periods.

*Effect of sensor device buoyancy on simulated mixing times*

Figure 2 shows mixing times predicted from tracer simulations using compartment models generated based on data from sensor devices with densities of ρ_p_ = 850 kg/m^3^, ρ_p_ = 900 kg/m^3^ and ρ_p_ = 950 kg/m^3^. The results demonstrate that the simulated mixing times are not particularly sensitive to the sensor device density.


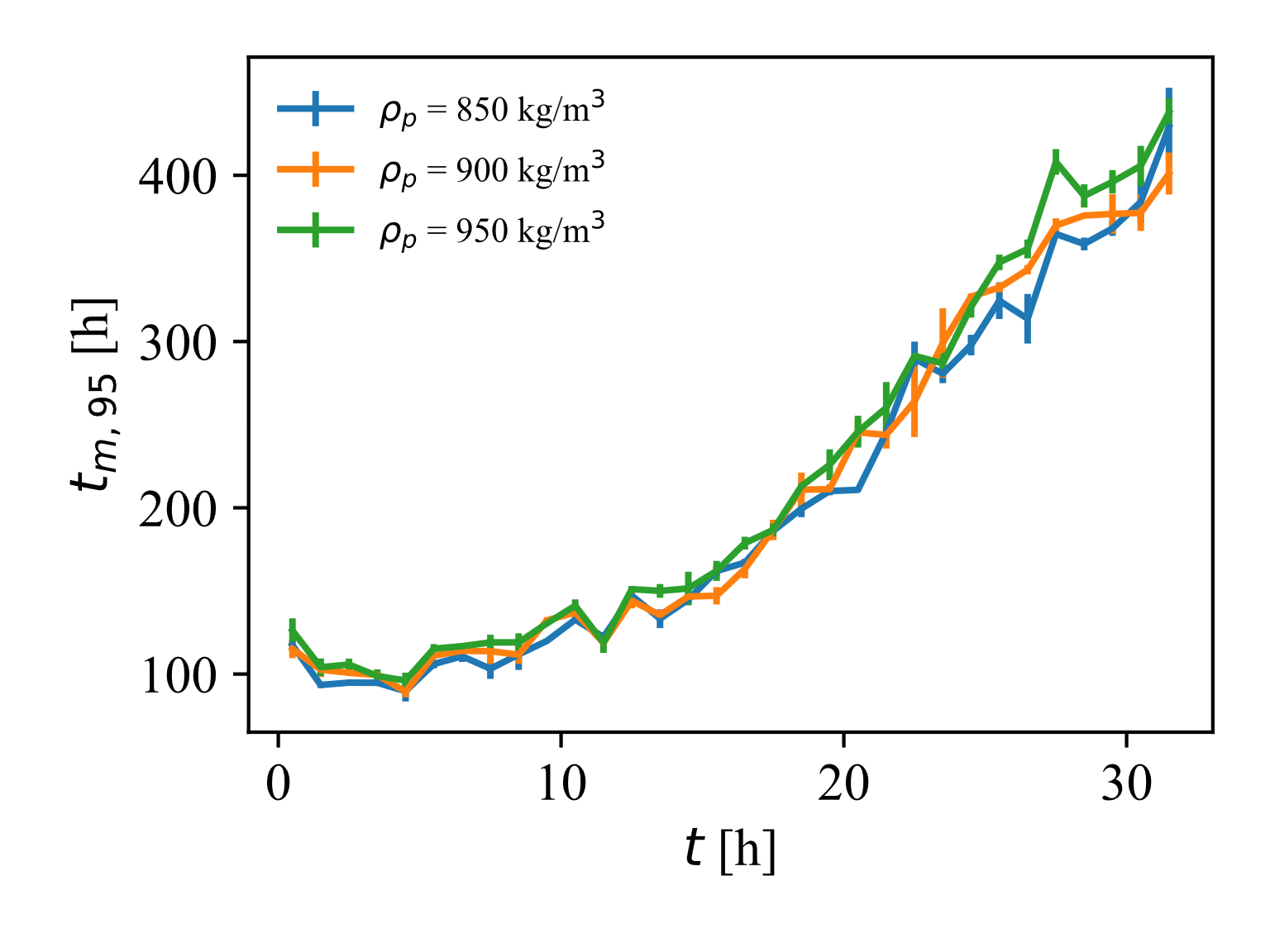


Figure 2. Simulated mixing times with compartment models generated based on three different sensor device densities (ρ_p_ = 850 kg/m^3^, ρ_p_ = 900 kg/m^3^ and ρ_p_ = 950 kg/m^3^).

*Model parameters and initial conditions*

Table 1. The initial conditions and the parameters used in the simulations.

| **Parameter/variable** | **Value** | **Unit** |
| --- | --- | --- |
| *C_s0_* | 7.0 | kg m^-3^ |
| *C_x0_* | 4.5 | kg m^-3^ |
| *C_p0_* | 0.10 | kg m^-3^ |
| *C_o0_* | 0.0064 | kg m^-3^ |
| *µ* | 0.171 | h^-1^ |
| *K_s_* | 3.44 ∙ 10^-3^ | kg m^-3^ |
| *K_o_* | 1.11 ∙ 10^-5^ | kg m^-3^ |
| *K_p_* | 115 | kg m^-3^ |
| *Y_px_* | 5.68 | kg kg^-1^ |
| *Y_xs_* | 0.761 | kg kg^-1^ |
| *Y_ps_* | 0.824 | kg kg^-1^ |
| *Y_so_* | 13.6 | kg kg^-1^ |
| *r_m,s_* | 0.0560 | kg kg^-1^ h^-1^ |
| *r_m,o_* | 0.0307 | kg kg^-1^ h^-1^ |
| *r_x,p_* | 0.284 | kg kg^-1^ h^-1^ |
